# Dihomo-γ-linolenic acid inhibits xenograft tumor growth in mice bearing shRNA-transfected HCA-7 cells targeting delta-5-desaturase

**DOI:** 10.1186/s12885-018-5185-9

**Published:** 2018-12-19

**Authors:** Yi Xu, Xiaoyu Yang, Di Gao, Liu Yang, Keith Miskimins, Steven Y. Qian

**Affiliations:** 1 Department of Pharmaceutical Sciences, North Dakota State University, Fargo, ND 58105 USA; 2 Department of Transplantation, Mayo Clinic Florida, Jacksonville, FL 32224 USA; 3 Cancer Biology Research Center, Sanford Research, Sioux Falls, SD 57104 USA

**Keywords:** COX-2-catalyzed DGLA peroxidation, Knockdown of delta-5-desaturase, Xenograft tumor, Cancer growth and migration, HDAC inhibitor

## Abstract

**Background:**

We previously demonstrated that knockdown of delta-5-desaturase via siRNA transfection together with dihomo-γ-linolenic acid supplementation inhibited colon cancer cell growth and migration, by promoting the production of the anti-cancer byproduct 8-hydroxyoctanoic acid from Cyclooxygenase-2-catalyzed dihomo-γ-linolenic acid peroxidation. Here, we extend our study to investigate the effects of delta-5-desaturase-knockdown and the resulting intensified dihomo-γ-linolenic acid peroxidation in xenograft tumor mice model.

**Methods:**

Four-week old nude mice bearing the human colon cancer cell HCA-7/C29 vs. its delta-5-desaturase knockdown analog (via shRNA transfection) were subject to 4-week treatments of: vehicle control, dihomo-γ-linolenic acid supplementation, 5-Fluorouracil, and combination of dihomo-γ-linolenic acid and 5-Fluorouracil. Tumor growth was monitored during the treatment. At the endpoint, the mice were euthanized and the tumor tissues were collected for further mechanism analysis.

**Results:**

Delta-5-desaturase knockdown (shRNA) together with dihomo-γ-linolenic acid supplementation increased 8-hydroxyoctanoic acid production to a threshold level in xenograft tumors, which consequently induced p53-dependent apoptosis and reduced tumors significantly. The promoted 8-hydroxyoctanoic acid formation was also found to suppress the tumors’ metastatic potential via regulating MMP-2 and E-cadherin expressions. In addition, our in vivo data showed that delta-5-desaturase knockdown along with dihomo-γ-linolenic acid supplementation resulted in anti-tumor effects comparable to those of 5-Fluorouracil.

**Conclusions:**

We have demonstrated that our paradigm-shifting strategy of knocking down delta-5-desaturase and taking advantage of overexpressed Cyclooxygenase-2 in tumor cells can be used for colon cancer suppression. Our research outcome will lead us to develop a better and safer anti-cancer therapy for patients.

**Electronic supplementary material:**

The online version of this article (10.1186/s12885-018-5185-9) contains supplementary material, which is available to authorized users.

## Background

Cyclooxygenase (COX) is a lipid-peroxidizing enzyme responsible for metabolizing polyunsaturated fatty acids to produce various lipid-derived molecules [1–3]. With Cyclooxygenase-1 being the constitutive isoform, Cyclooxygenase-2, the inducible form, can be readily induced in response to various stimuli including cancer promoters [4–6]. Overexpression of Cyclooxygenase-2 is a common phenomenon in many types of cancers. For example, it is known to overexpress in 85% of colorectal cancers and to be associated with colon cancer development by catalyzing peroxidation of arachidonic acid (AA, a downstream ω-6 fatty acid) to produce Prostaglandin E2 (PGE2) [7–9]. Hence, suppressing Cyclooxygenase-2 via inhibitor molecules has been extensively studied as a complementary therapy for cancer treatment [10, 11]. However, Cyclooxygenase-2 inhibitors have normally resulted in limited clinical outcomes for cancer patients as Cyclooxygenase-2 can be readily induced by various stimuli in the cancer environment [4–6, 12]. In addition, Cyclooxygenase-2 inhibitors have been found to commonly cause gastrointestinal injury and cardiovascular side effects in patients [13–15].

The ω-6 s and ω-3 s are two essential classes of dietary fatty acids. The ω-3 s have been shown to possess some anti-cancer activity and used as dietary supplements for cancer prevention and treatment, partially due to their competition against arachidonic acid for Cyclooxygenase-2 [16–20]. However, the more abundant ω-6 s (the ratio of ω-6 s vs. ω-3 s is 10:1 to 30:1 in the western diet [21–23]) have not received much research attention in cancer treatment due to the pro-cancer activities derived from Cyclooxygenase-2-catalyzed arachidonic acid peroxidation. Unlike many other research labs focusing on Cyclooxygenase-2 inhibition and ω-3 dietary supplementation in cancer treatment, our lab aims to develop an entirely novel anti-cancer strategy based on two often overlooked aspects: the commonly overexpressed Cyclooxygenase-2 in cancer, and the inevitable and abundant ω-6 s in our daily diet, to be exploited and manipulated to control cancers.

Delta-5 desaturase (D5D) is the rate-limiting enzyme that converts upstream ω-6 dihomo-γ-linolenic acid (DGLA) to arachidonic acid. Our previous studies showed that delta-5-desaturase knockdown (via siRNA) in the human colon cancer cell line HCA-7 colony 29 (HCA-7/C29, cells that express Cyclooxygenase-2) could promote the production of an anti-cancer byproduct, 8-hydroxyoctanoic acid (8-HOA), from Cyclooxygenase-2-catalyzed dihomo-γ-linolenic acid peroxidation, and thus inhibit cancer cell growth and migration [24–27]. The promoted 8-hydroxyoctanoic acid was found to induce p53-dependent apoptosis and cause DNA damage via serving as a histone deacetylase (HDAC) inhibitor [26, 27]. We thus proposed and demonstrated that, instead of inhibiting Cyclooxygenase-2, the commonly overexpressed Cyclooxygenase-2 in cancer cells can be used to elevate the production of 8-hydroxyoctanoic acid and thus to control cancer development, shifting the paradigm of Cyclooxygenase-2 cancer biology.

In the present study, we have made the first effort to test our novel anti-cancer concept and strategy using xenograft tumor models in nude mice bearing shRNA-transfected HCA-7/C29 targeting delta-5-desaturase (D5D-*KD* tumors). We have demonstrated that dihomo-γ-linolenic acid supplementation elevated 8-hydroxyoctanoic acid production in an autocrine manner to a threshold level (> 0.3 μg/g) in delta-5-desaturase-*KD* tumors and therefore significantly suppressed tumor growth (~ 40% reduction vs. delta-5-desaturase-*WT* tumor control). Formation of 8-hydroxyoctanoic acid was also found to induce p53-dependent apoptosis, and inhibited the metastatic potential of delta-5-desaturase-*KD* tumors. In addition, dihomo-γ-linolenic acid supplementation along with delta-5-desaturase knockdown was able to greatly promote the efficacy of 5-FU in inhibiting tumor growth (~ 70% reduction vs. control).

Besides having promising outcomes for treatment of colon cancer, we have also demonstrated that dihomo-γ-linolenic acid, along with a genetic delta-5-desaturase knockdown strategy, can suppress the growth, migration, and invasion of many other cancer cells, including pancreatic cancer BxPC-3 [27, 28], breast cancer MDA-MB-231 and 4 T1 [29], lung cancer A549, liver cancer HepG2, and their associated xenograft tumors (unpublished research results). Our new strategy of making use of commonly overexpressed Cyclooxygenase-2 for anti-cancer purpose represents a paradigm shifting concept as it challenges the conventional Cyclooxygenase-2 inhibition strategy in cancer treatment. Our on-going research tasks include optimization of dose/duration of dihomo-γ-linolenic acid supplementation, development of a delivering system (e.g., nanoparticles) of delta-5-desaturase-siRNA to tumors, and discovery of effective delta-5-desaturase inhibitors, aiming to translating our new anti-cancer strategy to clinical settings in the near future.

## Methods

### Chemicals and materials

Dihomo-γ-linolenic acid (purity > 99%, used for in vitro experiments) was obtained from Nu-Chek-Prep (MN, USA). Analytical standard grades of arachidonic acid, dihomo-γ-linolenic acid, PGE2, arachidonic acid-d_8_, dihomo-γ-linolenic acid-d_6_, and PGE_2_-d_9_ as well as dihomo-γ-linolenic acid ethyl ester (used for in vivo supplements) were purchased from Cayman Chemical (MI, USA). 8-hydroxyoctanoic acid and 5-FU were acquired from Sigma-Aldrich (MO, USA). Crystal violet, pentafluorobenzyl bromide, diisopropylethylamine, HPLC-MS grade water, acetonitrile, acetic acid and methanol were obtained from VWR (PA, USA). A SampliQ Silica C18 ODS reverse phase SPE cartridge was obtained from Agilent Technology (CA, USA).

### Biological reagents

CelLytic™ lysis reagent, delta-5-desaturase primary antibody (Cat# SAB2100744, used as 1:100 for western blot) and X-tremeGENE HP DNA transfection reagent were acquired from Sigma-Aldrich (MO, USA). GlutaMAX™ Opti-MEM reduced serum medium, Pure Link™ HQ Mini Plasmid DNA Purification Kit, T-Per tissue protein extraction reagent, and NE-PER™ nuclear and cytoplasmic extraction reagents were bought from Thermo Fisher Scientific (MA, USA). Fetal bovine serum (FBS) and Dulbecco’s Modified Eagle’s Medium were obtained from VWR (PA, USA). Annexin V Apoptosis Detection Kits I were acquired from BD Pharmingen™ (NJ, USA). Primary antibodies for immunofluorescence studies, e.g.*,* Cyclooxygenase-2 (Cat# ab15191, used as 1:800), delta-5-desaturase (Cat# ab126706, used as 1:800), MMP-2 (Cat# ab37150, used as 1:800), E-cadherin (Cat# ab76055, used as 1:800), cleaved PARP (Cat# ab32064, used as 1:800), Ki-67 (Cat# ab15880, used as 1:500) were purchased from Abcam (MA, USA). All the antibodies are validated with multiple published references; this information can be found in the corresponding product pages. CF633 goat anti-rabbit IgG(H + L) (Cat# 20122, used as 1:250) and CF633 goat anti-mouse IgG(H + L) (Cat# 20120, used as 1:250) were purchased from Biotium (CA, USA). γH2AX primary antibody (Cat# A300-081A, used as 1:100) was purchased from Bethyl Laboratories (TX, USA). Primary antibodies for p53 (Cat# 9282, used as 1:100), acetyl histone H3 (Cat# 9649, used as 1:100), procaspase 9 (Cat# 9502, used as 1:200), β-actin (Cat# 4970, used as 1:200), and horseradish peroxidase-conjugated anti-rabbit IgG (Cat# 7074, used as 1:200) were bought from Cell Signaling (MA, USA). DNA oligos encoding delta-5-desaturase-targeted pre-shRNA were purchased from Integrated DNA Technologies (IA, USA). A BLOCK-iT Pol II miR RNAi Expression Vector Kit was purchased from Invitrogen (NY, USA).

### Cancer cell line

A human colon cancer cell line HCA-7 colony 29 was used in this study. The cell line was purchased in April 2013 from European Collection of Cell Cultures (Catalog No.:02091238), and was recently tested as free of mycoplasma contamination in December 2017 by IDEXX BioResearch (MO, USA). The cells were grown in Dulbecco’s Modified Eagle’s Medium supplemented with 10% FBS. Cells were cultured in an incubator with a 95% humidified atmosphere and 5% CO_2_ at 37 °C.

A stable delta-5-desaturase-*KD* HCA-7/C29 cell line was created via shRNA transfection for the xenograft tumor study. Briefly, two strands of DNA oligonucleotides encoding delta-5-desaturase-targeted shRNA were designed with BLOCK-iT™ RNAi Designer (www.invitrogen.com/rnai) and purchased from Integrated DNA Technologies with the following sequences: target strand, TGCTGTAATCATCCAGGCCAAGTCCAGTTTTGGCCACTGACTGACTGGACTTGCTGGATGATTA; and complementary strand, CCTGTAATCATCCAGCAAGTCCAGTCAGTCAGT GGCCAAAACTGGACTTGGCCTGGATGATTAC. The delta-5-desaturase-targeted shRNA was then cloned into pcDNA^tm^ 6.2-GW/miR vector and transformed into *E.coli*. The plasmid DNA from the expression clone was extracted and transfected into wild type HCA-7/C29 cells for 24 h. For antibiotic selection, the cells were incubated in fresh complete medium containing 10 μg/ml of Blasticidin. The Blasticidin-containing medium was refreshed every 3–4 days until Blasticidin-resistant colonies were identified (~ 10–14 days). About 20 Blasticidin-resistant colonies were collected and expanded, followed by western blot analysis to evaluate the knockdown effect. A colony formation assay was conducted in order to determine whether shRNA-transfection affected the growth of HCA-7/C29 cells.

### Xenograft tumor model and mouse treatment

Four-week old female nude mice (J:Nu, stock number 007850) were purchased from The Jackson Laboratory (Bar Harbor, ME), and were housed in a pathogen-free Innovive IVC system with water and food ad libitum. After allowing the mice to acclimatize for 1 week, tumor xenografts were established by subcutaneously injecting 2 × 10^6^ delta-5-desaturase-*WT* or delta-5-desaturase-*KD* (shRNA) HCA-7/C29 cells into the hind flank of each mouse. The mice were then fed with a standard diet for two more weeks to allow the tumors to grow, and further divided into four sub-groups for four-week treatments (6 mice per groups): (1) vehicle control; (2) dihomo-γ-linolenic acid ethyl ester at a dose of 8 mg/mouse (in 250 μL 32% ethanol solution), oral gavage, twice a week; (3) 5-FU at 30 mg/kg (in 50 μL PBS), *i.v.* injection, twice a week; and (4) combination of dihomo-γ-linolenic acid ethyl ester and 5-FU. All animal experiments were approved by the Institutional Animal Care and Use Committee at North Dakota State University.

### Tumor size measurement

Tumor size was measured twice a week using a digital caliper during the entire treatment period. Tumor volume was calculated as: V = L × W^2^/2. After four-week treatment, the mice were euthanized to collect tumor tissues for further analysis described in following sections.

### Colony formation assay and in vitro apoptosis analysis

Cancer cell survival and apoptosis after various treatments were assessed by a colony formation assay and an Annexin V Apoptosis Detection Kit I, respectively, as described elsewhere [25–28]. In the colony formation assay in vitro, survival fraction = plating efficiency in treatment group/plating efficiency in control group. An Accuri C6 flow cytometer was used for apoptosis analysis (10,000 cells were counted for each sample). Unstained cells and cells stained with FITC Annexin V only or PI only were used to set up compensation and quadrants. Data were analyzed by FlowJo software.

### Western blot analysis

Expression of p53, procaspase 9, γH2AX and acetyl histone 3 in HCA-7/C29 and its delta-5-desaturase-*KD* analog upon different treatments in vitro were assessed by western blot as described elsewhere [24–30]. For their expressions in vivo*,* proteins from tumor tissues (~ 50–100 mg) were extracted followed by standard western blot procedures [24–30].

### Quantification of DGLA/AA ratio and PGE2 level

The amount of dihomo-γ-linolenic acid, arachidonic acid, and PGE2 in cells were quantified via LC/MS analysis as described elsewhere [24, 25]. For the in vivo study, tumor tissues were frozen in liquid nitrogen, crushed to a fine powder, and mixed with water, methanol, and internal standards (arachidonic acid-d_8_, dihomo-γ-linolenic acid-d_6_, and PGE2-d_9_). The mixtures were vortexed for 1 min and set on ice for 30 min, followed by the same extraction procedures and LC/MS analysis method as described in the in vitro experiment [24, 25].

### GC/MS analysis of 8-HOA

GC/MS analysis was employed to determine the amount of 8-hydroxyoctanoic acid (in its derivative of pentafluorobenzyl bromide) formed from cells after various treatments as described elsewhere [26, 28–30]. In the in vivo study, tumor tissues were frozen in liquid nitrogen, crushed to a fine powder, and mixed with water, methanol, HCl, an internal standard (hexanoic acid), and dichloromethane, followed by the same extraction and GC/MS analysis procedures as the in vitro experiment [26, 28–30].

### Immunofluorescence analysis

Immunofluorescence studies were performed to assess the expressions of delta-5-desaturase, Cyclooxygenase-2, cleaved PARP, Ki-67, MMP-2 and E-cadherin in tumor tissues as described elsewhere [30]. Briefly, tumor tissues were fixed with formaldehyde and embedded in paraffin blocks. Tissue sections were deparaffinized with xylene, rinsed, and rehydrated through a graded series of alcohol. For antigen retrieval, the slides were placed in a rack in the retriever (Aptum Biologics Ltd., UK) filled with sodium citrate buffer, the retriever was run for 30 min at preset pressure and temperature. Then the tumor sections were incubated with primary antibodies and secondary antibodies. Cell nuclei were counter-stained with DAPI. The images were acquired with a Zeiss Axio Imager M2 microscope.

### Statistics

All the quantification data were presented as mean ± standard deviation (SD) from at least three separate experiments (for in vitro studies), or from six tumor samples per treatment group (for in vivo studies). Statistical differences between groups were evaluated by analysis of variance and post hoc t-test; differences were considered significant with a *p*-value < 0.05.

## Results

### Formation of 8-HOA via COX-2-catalyzed DGLA peroxidation inhibits cancer cell growth

**Fig. 1:**
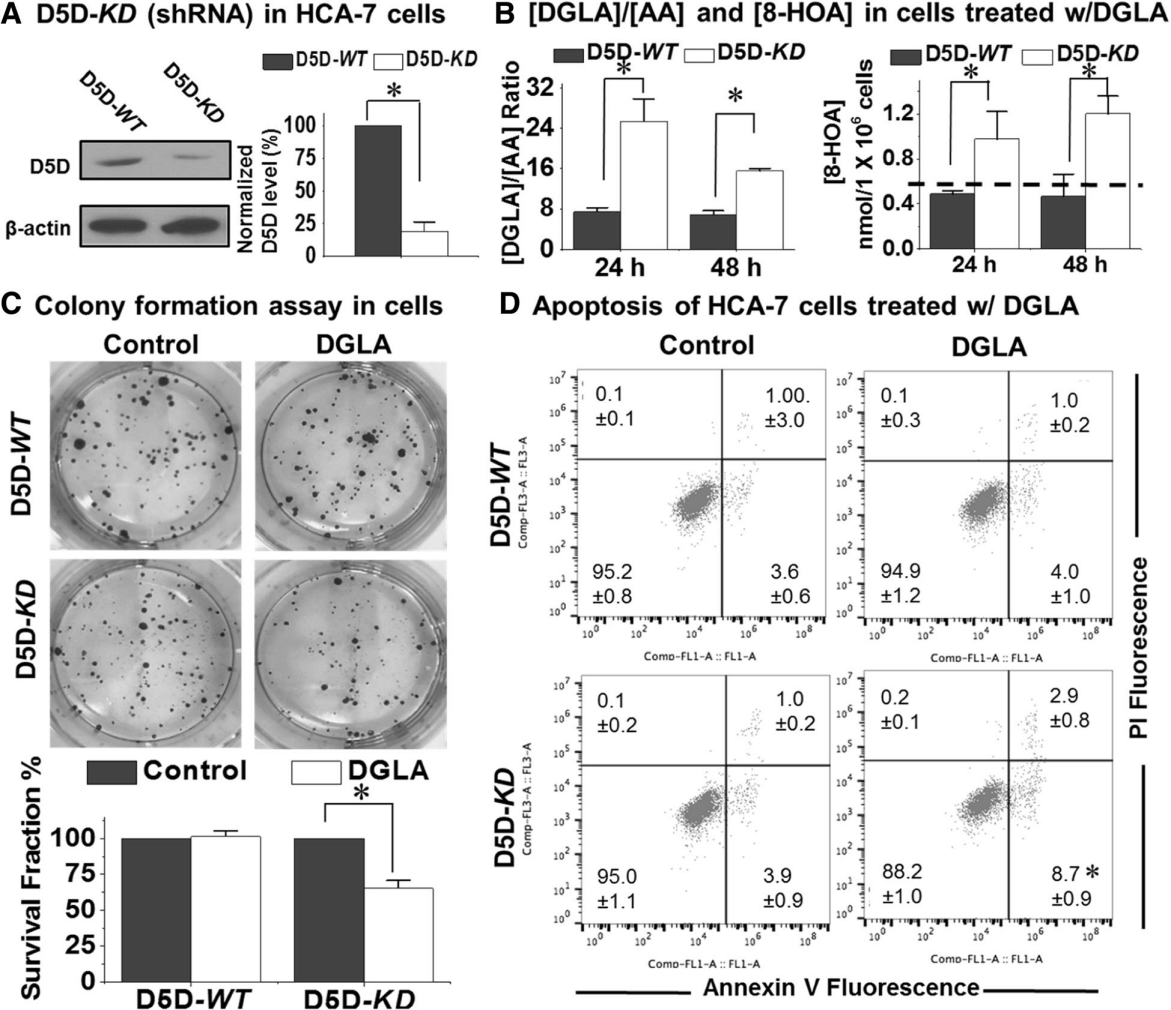
Treatment of dihomo-γ-linolenic acid elevates 8-hydroxyoctanoic acid production and suppresses cell growth in delta-5-desaturase-*KD* HCA-7 cells. **a** Western blotting of delta-5-desaturase expression level in HCA-7 cells with or without delta-5-desaturase shRNA transfection. The expression level of delta-5-desaturase was normalized using β-actin as a loading control; **b** Quantification of dihomo-γ-linolenic acid/arachidonic acid ratio (Left) and 8-hydroxyoctanoic acid level (Right) from 1.0 × 10^6^ of delta-5-desaturase-*WT* and delta-5-desaturase-*KD* HCA-7 cells after 24 h and 48 h incubation with 100 μM dihomo-γ-linolenic acid. The dashed line indicates the threshold level of 8-hydroxyoctanoic acid that can only be reached in delta-5-desaturase-*KD* cells; **c** Colony formation assay of delta-5-desaturase-*WT* and delta-5-desaturase-*KD* HCA-7 cells after exposed to 100 μM dihomo-γ-linolenic acid treatment for 48 h followed by additional 10 days incubation. The survival fractions for control groups were normalized to 100%; **d** Annexin V-FITC/PI staining of delta-5-desaturase-*WT* and delta-5-desaturase-*KD* HCA-7 cells after 48 h incubation with 100 μM dihomo-γ-linolenic acid. All of the data represent mean ± standard deviation with *n* ≥ 3. *: significant difference vs. control with *p* < 0.05

HCA-7/C29 is an epithelial type human colon carcinoma cell line, featuring high Cyclooxygenase-2 expression [31, 32]. Here, we created a stable delta-5-desaturase-knockdown HCA-7/C29 cells (D5D-*KD* cells) by shRNA-transfection, which yielded a ~ 75% suppressed delta-5-desaturase expression compared to that of delta-5-desaturase-wild type HCA-7/C29 cells (D5D-*WT* cells, Fig. 1a). Consistent with our previous siRNA experiments [25–27], a treatment with dihomo-γ-linolenic acid (100 μM) for 48 h in delta-5-desaturase*-KD* cells led to a significantly elevated ratio of dihomo-γ-linolenic acid/arachidonic acid, which resulted in increased 8-hydroxyoctanoic acid production from dihomo-γ-linolenic acid peroxidation to a threshold level (> 0.5 nmol/10^6^ cells, dashed line in Fig. 1b, [25–29]). However, the ratio of dihomo-γ-linolenic acid/arachidonic acid in delta-5-desaturase-*WT* cells with dihomo-γ-linolenic acid treatment was significantly lower, therefore, the 8-hydroxyoctanoic acid production was unable to reach the threshold level in these cells as both dihomo-γ-linolenic acid and arachidonic acid serve as the substrates that compete for Cyclooxygenase-2-catalyzed peroxidation.

Results revealed that the elevated 8-hydroxyoctanoic acid production from dihomo-γ-linolenic acid treatment significantly suppressed colony formation in delta-5-desaturase*-KD* cells (the surviving fraction was 63.2 ± 4.1% vs. 100% in control). In comparison, dihomo-γ-linolenic acid treatment did not lead to any inhibitory effect on delta-5-desaturase-*WT* cells (Fig. 1c). Induced apoptosis was also noted in delta-5-desaturase*-KD* cells with dihomo-γ-linolenic acid treatment, as demonstrated by annexin V-FITC/PI staining (population of early apoptotic cells 8.7 ± 0.9% vs. 3.9 ± 0.9% in the control, Fig. 1d). Again, dihomo-γ-linolenic acid treatment did not result in apoptosis in delta-5-desaturase-*WT* cells due to the low 8-hydroxyoctanoic acid production. It is noteworthy that delta-5-desaturase-*KD* treatment alone (i.e., without dihomo-γ-linolenic acid treatment) had no influence on HCA-7/C29 cell growth (Additional file 1: Figure S1).

### DGLA enhances 5-FU efficacy in D5D-*KD* cells

**Fig. 2:**
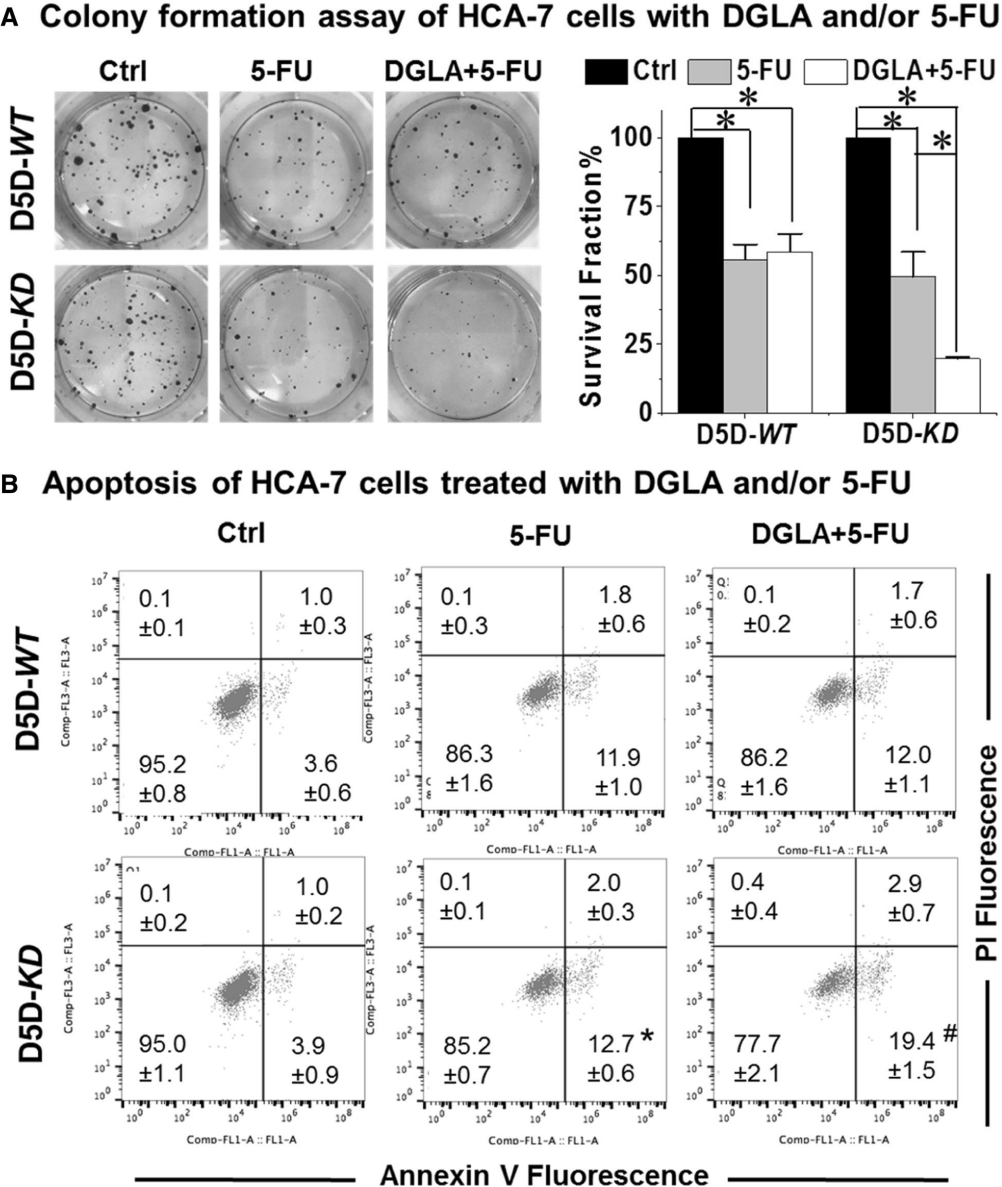
Dihomo-γ-linolenic acid treatment improves 5-FU’s efficacy in delta-5-desaturase-*KD* HCA-7 cells. **a** Colony formation assay of delta-5-desaturase-*WT* and delta-5-desaturase-*KD* HCA-7 cells after exposed to 100 μM dihomo-γ-linolenic acid and/or 50 μM 5-FU treatment for 48 h followed by additional 10 days incubation. *: significant difference with *p* < 0.05; **b** Annexin V-FITC/PI staining of delta-5-desaturase-*WT* and delta-5-desaturase-*KD* HCA-7 cells after 48 h incubation with 100 μM dihomo-γ-linolenic acid and/or 50 μM 5-FU. *: significant difference vs. control with *p* < 0.05, #: significant difference vs. 5-FU only group with *p* < 0.05. All of the data represent mean ± standard deviation with *n* ≥ 3

Chemo-resistance remains one of the major issues in cancer treatment [33]. Combinations of 5-FU with various other cancer therapeutic agents as well as fatty acid supplementation have been extensively studied to enhance the efficacy of 5-FU against cancer cells [34–37]. Here we observed that concurrent treatment with dihomo-γ-linolenic acid (100 μM) in delta-5-desaturase-*KD* cells could enhance 5-FU’s cytotoxicity with a survival fraction of 19.2 ± 1.5% vs. 48.1 ± 9.7% from 5-FU treatment (50 μM) alone (Fig. 2a). Consistently, co-treatment of dihomo-γ-linolenic acid promoted 5-FU-induced apoptosis in delta-5-desaturase-*KD* cells with early apoptotic cell population of 19.4% ± 1.5% vs. 12.7% ± 0.6% from 5-FU treatment alone (3.9% ± 0.9% in control, Fig. 2b). However, dihomo-γ-linolenic acid treatment did not improve 5-FU’s cytotoxicity on delta-5-desaturase-*WT* cells due to the low level of 8-hydroxyoctanoic acid production (Fig. 1b).

### DGLA supplementation promotes production of 8-HOA in D5D-*KD* xenograft tumors

Xenograft tumors were established by injecting HCA-7/C29 cells or their delta-5-desaturase-*KD* counterpart subcutaneously into the hind flank of each mouse. The mice were then subjected to 4-week treatments of 1) vehicle control, 2) oral gavage with dihomo-γ-linolenic acid ethyl ester, 3) *i.v.* injection of 5-FU, and 4) a combination of dihomo-γ-linolenic acid ethyl ester and 5-FU.

**Fig. 3:**
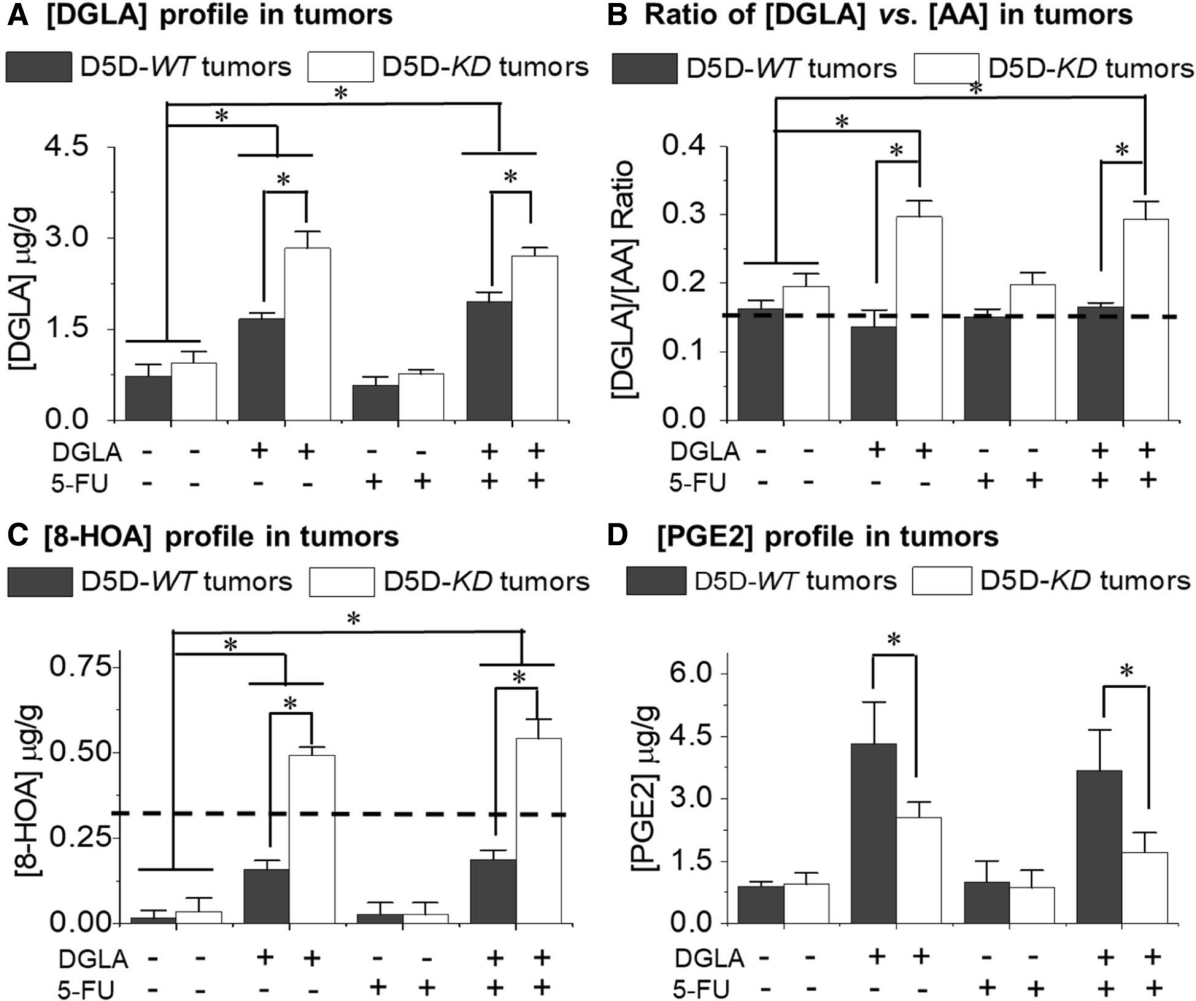
Delta-5-desaturase knockdown leads to accumulation of dihomo-γ-linolenic acid and 8-hydroxyoctanoic acid in xenograft tumor tissues. **a** LC/MS profile of dihomo-γ-linolenic acid levels in all groups of tumor tissues; **b** Calculated dihomo-γ-linolenic acid/arachidonic acid ratio from LC/MS analysis in different tumor tissues. The dashed line represents the basal level of dihomo-γ-linolenic acid/arachidonic acid ratio in tumors from mice receiving no dihomo-γ-linolenic acid supplementation; **c** GC/MS profile of 8-hydroxyoctanoic acid production from delta-5-desaturase-*WT* and delta-5-desaturase-*KD* tumor tissues. The dashed line represents the threshold level of 8-hydroxyoctanoic acid that can be only reached in delta-5-desaturase-*KD* tumors; **d** LC/MS profile of PGE2 from delta-5-desaturase-*WT* and delta-5-desaturase-*KD* tumor tissue. All of the data represent mean ± standard deviation with *n* = 6. *: significant difference with *p* < 0.05

Data from HPLC/MS analysis showed that, in the tumors from the mice without dihomo-γ-linolenic acid supplementation, only basal levels of dihomo-γ-linolenic acid were detected (~ 0.6 to 0.9 μg/g, Fig. 3a) and ratios of dihomo-γ-linolenic acid/arachidonic acid ranged from ~ 0.15 to 0.2 (Fig. 3b). In comparison, in the mice that received dihomo-γ-linolenic acid supplementation, we observed significantly elevated dihomo-γ-linolenic acid level in delta-5-desaturase-*WT* tumors (~ 1.7 to 1.9 μg/g, Fig. 3a); however, the dihomo-γ-linolenic acid/arachidonic acid ratio still remained similar to the basal level (~ 0.14 to 0.16, Fig. 3b) due to the effective conversion of dihomo-γ-linolenic acid to arachidonic acid in delta-5-desaturase-*WT* tumors. Most importantly, in delta-5-desaturase-*KD* tumors from mice with dihomo-γ-linolenic acid supplementation, we observed the most significantly increased dihomo-γ-linolenic acid level (~ 2.8 μg/g, Fig. 3a), while the ratio of dihomo-γ-linolenic acid/arachidonic acid also became greatly elevated compared to other groups (~ 0.30, double the basal level, dashed line in Fig. 3b) due to the suppressed conversion of dihomo-γ-linolenic acid to arachidonic acid.

GC/MS data revealed that dihomo-γ-linolenic acid supplementation resulted in significantly elevated 8-hydroxyoctanoic acid levels (~ 0.5 μg/g) in delta-5-desaturase-*KD* tumors vs. delta-5-desaturase-*WT* tumors (< 0.19 μg/g, Fig. 3c), while only basal levels of 8-hydroxyoctanoic acid were detected in the mice without dihomo-γ-linolenic acid supplementation despite the lower delta-5-desaturase expression levels (< 0.04 μg/g, Fig. 3c). In addition, consistent with the suppressed conversion of dihomo-γ-linolenic acid to arachidonic acid in delta-5-desaturase-*KD* tumors, the PGE2 levels in delta-5-desaturase-*KD* tumors were significantly lower than those in delta-5-desaturase-*WT* tumors from mice with dihomo-γ-linolenic acid supplementation (~ 2.0 vs. ~ 4.0 μg/g, Fig. 3d).

### Increased levels of 8-HOA lead to suppression of xenograft tumor growth

**Fig. 4:**
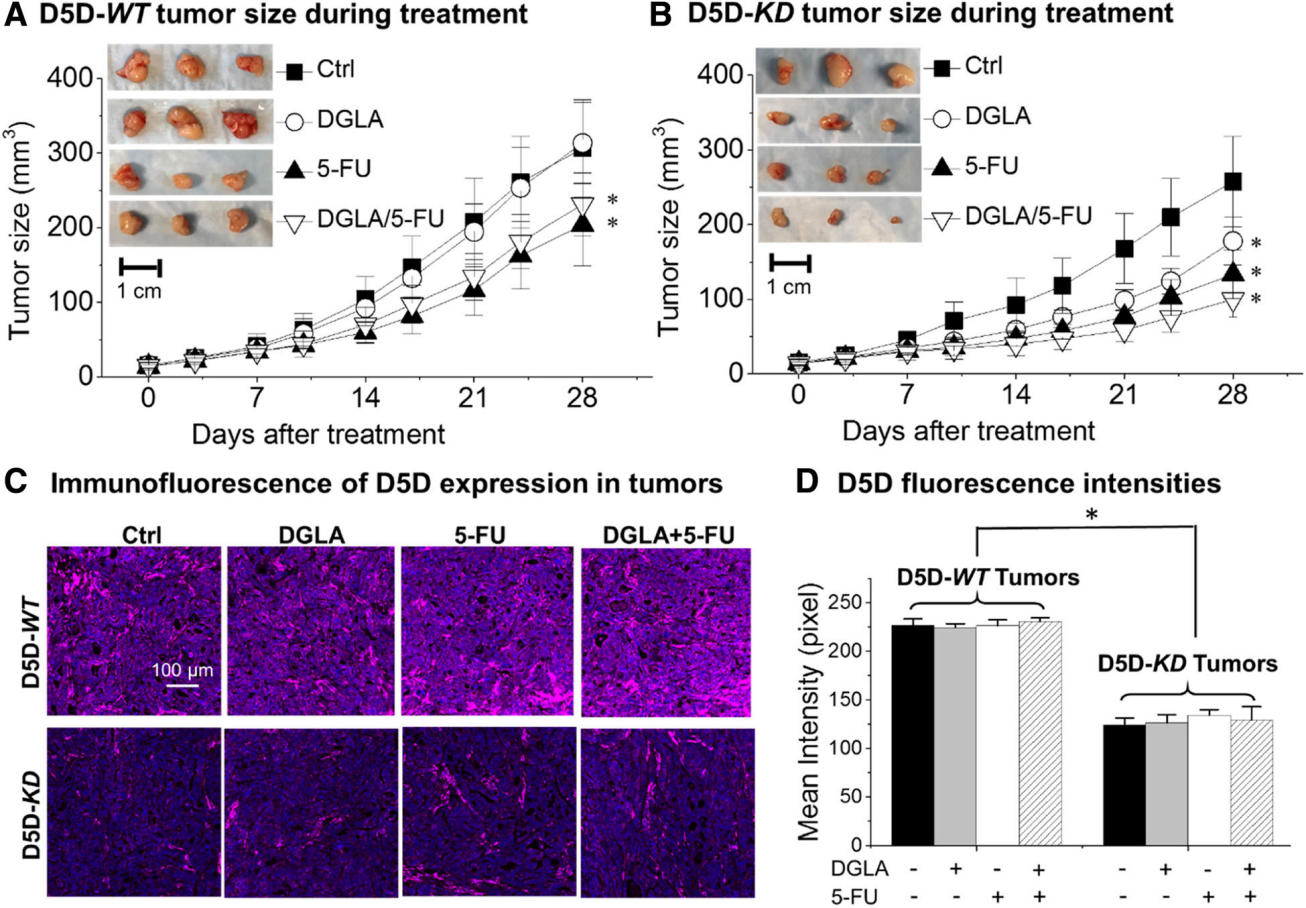
Promoted 8-hydroxyoctanoic acid inhibited growth of HCA-7 xenograft tumor. **a** Tumor size measurement of delta-5-desaturase-*WT* tumors during 4-week treatment of vehicle control, dihomo-γ-linolenic acid, 5-FU, or dihomo-γ-linolenic acid+ 5-FU. Insert: representative tumor photos from each treatment group at the end of the treatment; **b** Tumor size measurement of delta-5-desaturase-*KD* tumors during 4-week treatment. Insert: representative tumor photos from each treatment group at the end of the treatment; *: significant difference vs. corresponding control with *p* < 0.05; **c** Representative immunofluorescence images for delta-5-desaturase expression in tumor tissues; delta-5-desaturase was stained in pink, cell nuclei were counter stained with DAPI; **d** Quantification of the mean intensity of delta-5-desaturase staining in each sample. All of the data represent mean ± standard deviation with *n* = 6. *: significant difference with *p* < 0.05

Tumor size measurements showed that 4 weeks of dihomo-γ-linolenic acid supplementation had no significant effect on the growth of delta-5-desaturase-*WT* tumors (Fig. 4a). By comparison, dihomo-γ-linolenic acid supplementation significantly decreased the sizes of delta-5-desaturase-*KD* tumors relative to the control group (Fig. 4b), associated with the elevated 8-hydroxyoctanoic acid production. It was noted that about a 40% size reduction was achieved in delta-5-desaturase-*KD* tumors in mice treated with dihomo-γ-linolenic acid supplementation vs. delta-5-desaturase-*WT* tumors in mice treated with vehicle control (Fig. 4a).

Data also showed that 5-FU was able to inhibit tumor growth in both the delta-5-desaturase-*WT* group and the delta-5-desaturase-*KD* group (Fig. 4a and b). It is noteworthy that dihomo-γ-linolenic acid supplementation resulted in an average tumor size of ~ 178.2 ± 31.9 mm^3^ in delta-5-desaturase-*KD* tumors (Fig. 4b), leading to a similar effect compared to 5-FU treatment in delta-5-desaturase-*WT* tumors (~ 204.3 ± 55.3 mm^3^, Fig. 4a). In addition, concurrent treatment with dihomo-γ-linolenic acid along with 5-FU in mice bearing delta-5-desaturase-*KD* tumors led to an improved tumor size reduction (100.1 ± 24.3 mm^3^, Fig. 4b).

In order to validate shRNA knockdown efficiency during the 4-week treatment, immunofluorescence studies were performed to assess the expression levels of delta-5-desaturase in tumor tissues. Data revealed that delta-5-desaturase*-KD* tumors had significant lower delta-5-desaturase expressions than delta-5-desaturase-*WT* tumors for all treatments (Fig. 4c and d).

### Effect of promoted 8-HOA on tumor proliferation and apoptosis

**Fig. 5:**
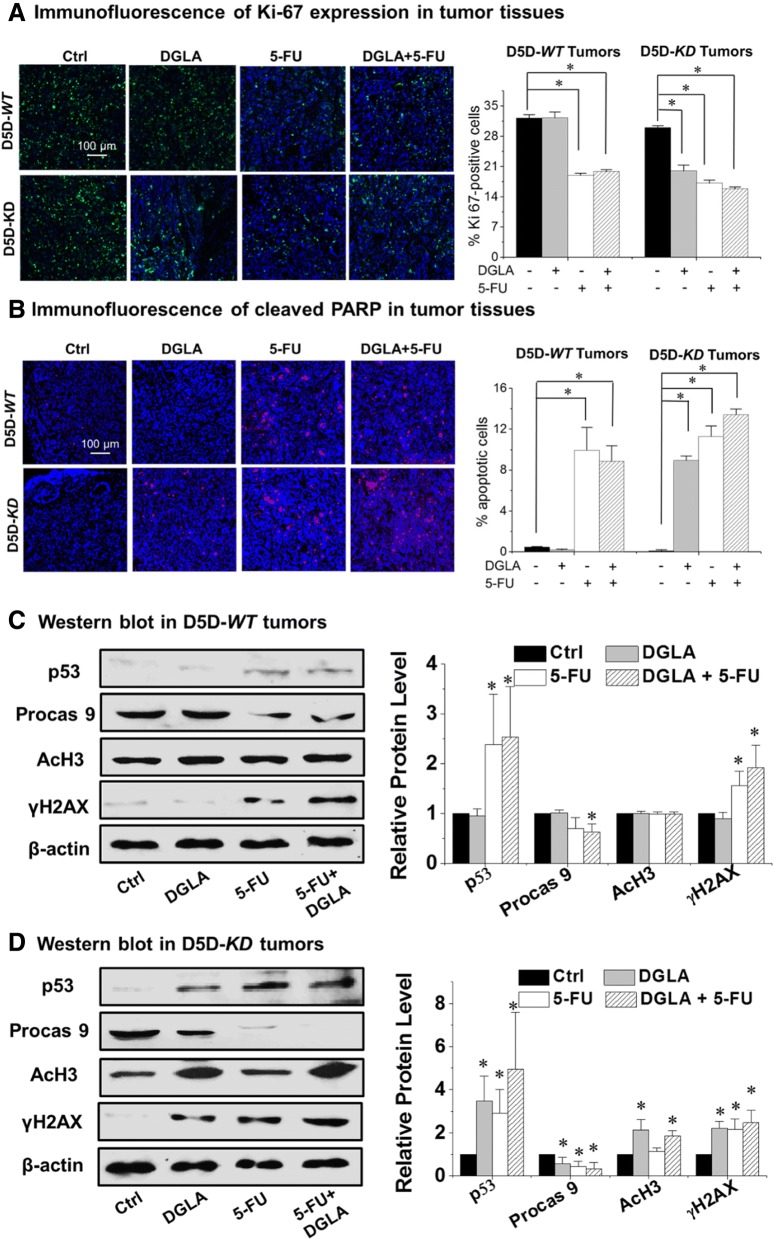
Expressions of apoptosis and proliferation-related proteins in tumor tissues. **a** Left panel, representative fluorescence images for Ki-67 expression in tumor tissues. Ki-67 were stained in green, cell nuclei were counter stained with DAPI. Right panel, quantification analysis of Ki-67; the results are presented as the percentage of Ki-67 positive cells to the total number of cells in each image; **b** Left panel, representative fluorescence images for cleaved PARP expression in tumor tissues. Cleaved PARP expression were stained in red, and cell nuclei were counter stained with DAPI. Right panel, quantification analysis of cell apoptosis; the results are presented as the percentage of cleaved PARP positive cells to the total number of cells in each sample. All of the data represent mean ± standard deviation with *n* = 6. *: significant difference with *p* < 0.05. **c** and **d** Western blotting of p53, procaspase 9, acetyl histone H3 and γH2AX in delta-5-desaturase-*WT* and delta-5-desaturase-*KD* tumor tissues.The relative expression level of each protein was normalized using β-actin as a loading control; All of the data represent mean ± standard deviation with *n* = 6. *: significant difference vs. control with *p* < 0.05

The expression of Ki-67 in tumor tissues was detected by immunofluorescence to assess tumor proliferation. Data showed that, dihomo-γ-linolenic acid supplementation led to significantly less Ki-67 expression in delta-5-desaturase-*KD* tumors, i.e., the percentage of Ki-67-positive cells was 19.9 ± 1.4% vs. 30.0 ± 0.5% in the control (Fig. 5a), whereas dihomo-γ-linolenic acid supplementation alone did not alter Ki-67 expression in delta-5-desaturase-*WT* tumors. In addition, while treatment with 5-FU alone suppressed tumor proliferation in both delta-5-desaturase-*WT* and delta-5-desaturase-*KD* tumors, the combination of dihomo-γ-linolenic acid supplementation and 5-FU in delta-5-desaturase-*KD* tumors resulted in slightly less Ki-67 expression.

Tumor cell apoptosis was assessed by immunostaining of cleaved PARP (a tumor apoptotic marker). Data revealed that dihomo-γ-linolenic acid supplementation only induced apoptosis in delta-5-desaturase-*KD* tumors, while having no such effect in delta-5-desaturase-*WT* tumors (Fig. 5b). In addition, while treatment with 5-FU alone induced apoptosis in both delta-5-desaturase-*WT* and delta-5-desaturase-*KD* tumors, the combination of dihomo-γ-linolenic acid supplementation and 5-FU in delta-5-desaturase-*KD* tumors caused more apoptosis than their individual treatments did (Fig. 5b).

Western blotting analysis revealed that dihomo-γ-linolenic acid supplementation did not alter the expression of apoptotic proteins in delta-5-desaturase-*WT* tumors (Fig. 5c). By comparison, in delta-5-desaturase-*KD* tumors, dihomo-γ-linolenic acid supplementation significantly increased the expression of p53 and decreased the expression of procaspase 9, indicating the activation of the p53-dependant apoptotic pathway (Fig. 5d). We also observed that dihomo-γ-linolenic acid supplementation caused the up-regulation of acetyl histone H3 and γH2AX in delta-5-desaturase-*KD* tumors (Fig. 5d), which is consistent with our previous reports in siRNA-transfected delta-5-desaturase cells [25–27]. These data together suggested that the elevated endogenous 8-hydroxyoctanoic acid production in delta-5-desaturase-*KD* tumors could suppress tumor growth, likely via affecting histone acetylation/deacetylation and causing DNA damage.

### Elevated 8-HOA production suppresses metastasis potential in D5D-*KD* tumors

**Fig. 6:**
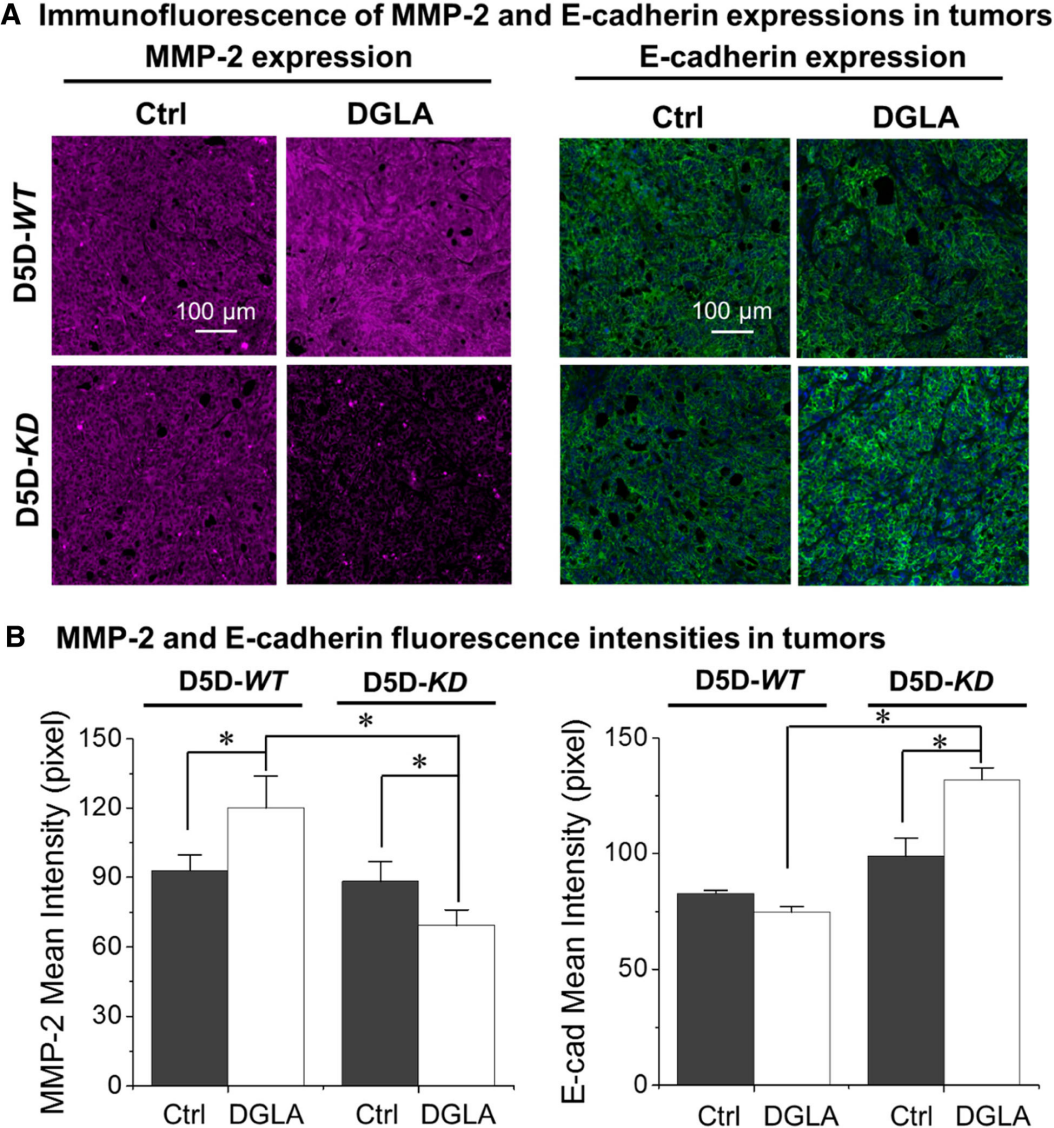
dihomo-γ-linolenic acid supplementation suppresses the metastasis potential in delta-5-desaturase-*KD* tumor tissues. **a** Representative images for MMP-2 and E-cadherin expressions in tumor tissues. MMP-2 was stained in red, E-cadherin was stained in green, cell nuclei were counter stained with DAPI; and **b** Mean intensities of MMP-2 and E-cadherin in each sample. All of the data represent mean ± standard deviation with *n* = 6. *: significant difference with *p* < 0.05

Data from immunofluorescence studies showed that dihomo-γ-linolenic acid supplementation increased the expression of MMP-2 (a marker for tumor metastasis) in delta-5-desaturase-*WT* tumors, while significantly decreasing MMP-2 expression in delta-5-desaturase*-KD* tumors (Fig. 6a and b). Consistently, dihomo-γ-linolenic acid supplementation also increased the expression of E-cadherin (a tumor metastasis inhibitor) in delta-5-desaturase-*KD* tumors compared to the vehicle control, while no such effect was observed in delta-5-desaturase-*WT* tumor tissues.

## Discussion

We had previously demonstrated that siRNA-delta-5-desaturase knockdown in different types of cancer cells can promote the production of 8-hydroxyoctanoic acid from intensified Cyclooxygenase-2-catalyzed dihomo-γ-linolenic acid peroxidation; the 8-hydroxyoctanoic acid served as an HDAC inhibitor to suppress cancer cell growth, migration, and invasion [25–29]. In the present study, we created stable delta-5-desaturase-*KD* HCA-7/C29 cells via shRNA transfection and made the first effort to test the anti-tumor effect of our novel strategy in xenograft tumors.

Our data showed that shRNA knockdown of delta-5-desaturase in HCA-7/C29 cells promoted 8-hydroxyoctanoic acid production from dihomo-γ-linolenic acid peroxidation, which then significantly suppressed the growth of delta-5-desaturase-*KD* cells in vitro (Fig. 1). Western blot (data not shown) further confirmed that promoting 8-hydroxyoctanoic acid formation from Cyclooxygenase-2-catalyzed dihomo-γ-linolenic acid peroxidation in delta-5-desaturase-*KD* cells resulted in a significant increase of acetyl histone H3 and γH2AX. We demonstrated again that the anti-proliferation effect of dihomo-γ-linolenic acid is actually derived from 8-hydroxyoctanoic acid’s action to inhibit HDAC and damage DNA in cells [25–27]. In addition, our strategy of delta-5-desaturase knockdown and dihomo-γ-linolenic acid treatment also improved the cytotoxicity of 5-FU to cancer cells (Fig. 2).

Consistently, our in vivo data demonstrated that delta-5-desaturase knockdown in xenograft tumors led to elevated levels of 8-hydroxyoctanoic acid in mice with dihomo-γ-linolenic acid supplementation (Fig. 3), which consequently inhibited the tumor growth (Fig. 4, Additional file 2: Table S1 and Additional file 3: Table S2). In addition, while 5-FU treatment alone was able to suppress the growth of both delta-5-desaturase-*WT* and delta-5-desaturase-*KD* tumors (Fig. 4, Additional file 2: Table S1 and Additional file 3: Table S2), a two-factor analysis (considering 5-FU and delta-5-desaturase-*KD*/dihomo-γ-linolenic acid as the factors, Additional file 4: Table S3) suggested an additive effect on tumor inhibition from the combination of 5-FU and delta-5-desaturase-*KD*/dihomo-γ-linolenic acid. In our future studies, we plan to test the combinational effects of our strategy with different chemo-drugs, including decitabine and sorafenib, as they are reported to synergize with HDAC inhibitors to exert anti-cancer activities [38, 39].

We have also noted that, without dihomo-γ-linolenic acid supplementation, the tumor size of the delta-5-desaturase-*KD* vehicle control group is slightly smaller than that of the delta-5-desaturase-*WT* vehicle control group (Fig. 4), consistent with the relatively higher concentration of dihomo-γ-linolenic acid, ratio of dihomo-γ-linolenic acid/arachidonic acid, and concentration of 8-hydroxyoctanoic acid (although 8-hydroxyoctanoic acid never reached the threshold level). In the in vitro system, delta-5-desaturase-*KD* treatment alone *(*i.e.*,* without dihomo-γ-linolenic acid treatment) had no influence on HCA-7/C29 cell growth (Additional file 1: Figure S1). Since upstream ω-6 s in the diet may not be able to convert enough dihomo-γ-linolenic acid in the body, certain amount of dihomo-γ-linolenic acid supplementation is necessary to elicit its anti-cancer activities for reaching a threshold level of 8-hydroxyoctanoic acid from Cyclooxygenase-2 peroxidation.

The body weights of the all of experimental mice were monitored throughout the treatment period (Additional file 5: Figure S2), and no significant change was noted among the different treatment groups.

Results also showed that dihomo-γ-linolenic acid supplementation led to increased MMP-2 expression (i.e.*,* higher metastasis potential) and elevated levels of arachidonic acid as well as PGE2 (Fig. 3d) in delta-5-desaturase-*WT* tumors (Fig. 6). PGE2 has been shown to play a role in cancer migration [40, 41]. However, in delta-5-desaturase-*KD* tumors, dihomo-γ-linolenic acid supplementation suppressed MMP-2 expression, associated with higher levels of 8-hydroxyoctanoic acid and lower levels of PGE2 (Fig. 3). E-cadherin is a cell adhesion molecule; decreased E-cadherin expression in the tumor environment is correlated with a strong invasive potential [42]. Here we observed that dihomo-γ-linolenic acid supplementation greatly elevated E-cadherin levels in delta-5-desaturase-*KD* tumors compared to the vehicle control (Fig. 6), indicating less invasive potential. In this study, no spontaneously metastasizing tumor was observed in the subcutaneous xenograft model, therefore, our on-going research on orthotopic colon tumors will provide more insight into how our strategy would actually perform on metastasizing tumors and in cancer patients, as that model has a tumor microenvironment very similar to the original tumor.

It has been a challenge to deliver therapeutic RNAs to tumors due to various issues and concerns [43]. In our on-going study, we are employing innovative 3-way-junction RNA nanoparticles to specifically deliver delta-5-desaturase-targeting siRNA into cancer cells/tumors [44–46]. The newly developed multi-functional, thermodynamically and chemically stable RNA nanoparticles were designed to harbor cancer targeting ligands as well as delta-5-desaturase-targeted siRNA to inhibit delta-5-desaturase expression specifically in tumor cells. Our ongoing study has shown that the RNA nanoparticles carrying delta-5-desaturase-siRNA specifically targeting tumors are able to inhibit delta-5-desaturase expression and suppress colon cancer growth when dihomo-γ-linolenic acid is supplemented concurrently. In addition, it has been reported that various small compounds possess potent delta-5-desaturase inhibitory activities which can be potentially applied in our strategy for clinic use [47–49]. We are also working on developing new specific and effective delta-5-desaturase inhibitors for use in cancer patients (pending US-provisional patent application).

## Conclusion

The present research demonstrated that delta-5-desaturase knockdown and dihomo-γ-linolenic acid supplementation in HCA-7/C29 xenograft tumors results in elevated 8-hydroxyoctanoic acid production, which serves an HDAC inhibitor to induce cell apoptosis pathway and suppress tumor growth. Compared to the more conventional Cyclooxygenase-2 inhibition strategy, our novel strategy of inhibiting delta-5-desaturase and taking advantage of the high Cyclooxygenase-2 expression in cancer cells will lead to better anti-cancer effects in two ways: stimulating an anti-cancer effect from dihomo-γ-linolenic acid while decreasing the pro-cancer effect from arachidonic acid. In addition, considering the fact that cancer cells in general have overexpressed Cyclooxygenase-2 levels and higher fatty acid intake rates than normal cells and tissues [7–9, 50], we anticipate that our strategy will lead to fewer side effects and safer cancer treatment outcomes.

## Additional files

Additional file 1: **Figure S1.** D5D-*KD* alone did not affect HCA-7 cell growth. A. Colony formation of D5D-*WT* and D5D-*KD* HCA-7 cells at 10 days without DGLA treatment. B. Calculated plate efficiencies (i.e. total number of colonies counted/total number of cells seeded). (DOCX 248 kb)

Additional file 2: **Table S1.** Statistical analysis (ANOVA) for tumor size in Fig. 4a. The four groups are: Group 1: D5D-*WT* tumor control; Group 2: D5D-*WT* tumor w/DGLA; Group 3: D5D-*WT* tumor w/5-FU; Group 4: D5D-*WT* tumor w/DGLA and 5-FU. The six inserts (left to right from first row to second row) in each cell represents the statistics data at 10, 14, 17, 21, 24 and 28 days after treatment, respectively. *: significance with *p* < 0.05, **: significance with *p* < 0.01. (DOCX 16 kb)

Additional file 3: **Table S2.** Statistical analysis (ANOVA) for tumor size in Fig. 4b. The four groups are: Group 5: D5D-*KD* tumor control; Group 6: D5D-*KD* tumor w/DGLA; Group 7: D5D-*KD* tumor w/5-FU; Group 8: D5D-*KD* tumor w/DGLA and 5-FU. The six inserts (left to right from first row to second row) in each cell represents the statistics data at 10, 14, 17, 21, 24 and 28 days after treatment, respectively. *: significance with *p* < 0.05, **: significance with *p* < 0.01. (DOCX 16 kb)

Additional file 4: **Table S3.** Two factor experiment table of the additive effect from D5D-*KD*/DGLA along with 5-FU’s on HCA-7 xenograft tumor growth. Measured tumor size after 4-week treatment from (a) mice with D5D-*WT* tumor after vehicle treatment, (b) mice with D5D-*WT* tumor after 5-FU treatment, (c) mice with D5D-*KD* tumor after DGLA supplementation, and (d) mice with D5D-*KD* tumor after combination of DGLA and 5-FU treatment. Data represent mean ± SD with six mice per groups. (DOCX 15 kb)

Additional file 5: **Figure S2.** Body weight of mice bearing HCA-7 xenograft tumors during 4-week treatment. A. Measured body weight of mice bearing D5D-*WT* tumors during 4-week treatment. B. Measured body weight of mice bearing D5D-*KD* tumors during 4-week treatment. (DOCX 71 kb)

## Abbreviations

5-FU: 5-flurouracil
8-HOA: 8-hydroxyoctanoic acid
AA: Arachidonic acid
COX: Cyclooxygenase
D5D: Delta-5-desaturase
D5D-*KD*: Delta-5-desaturase knockdown
D5D-*WT*: Wild type D5D
DGLA: Dihomo-γ-linolenic acid
HCA-7/C29: HCA-7 colony 29 cells
HDAC: Histone deacetylase
PGE2: Prostaglandin E2
