# Supplementary material for: Dihomo-γ-linolenic acid inhibits xenograft tumor growth in mice bearing shRNA-transfected HCA-7 cells targeting delta-5-desaturase
Source: BMC Cancer. 2018 Dec 19;18:1268. doi: 10.1186/s12885-018-5185-9 (PMC6299961; doi:10.1186/s12885-018-5185-9)
Supplement: Supplementary file 1 — Figure S1. D5D-KD alone did not affect HCA-7 cell growth. A. Colony formation of D5D-WT and D5D-KD HCA-7 cells at 10 days without DGLA treatment. B. Calculated plate efficiencies (i.e. total number of colonies counted/total number of cells seeded). (DOCX 248 kb) [file 12885_2018_5185_MOESM1_ESM.docx]

**Supplemental Figure 1**

**
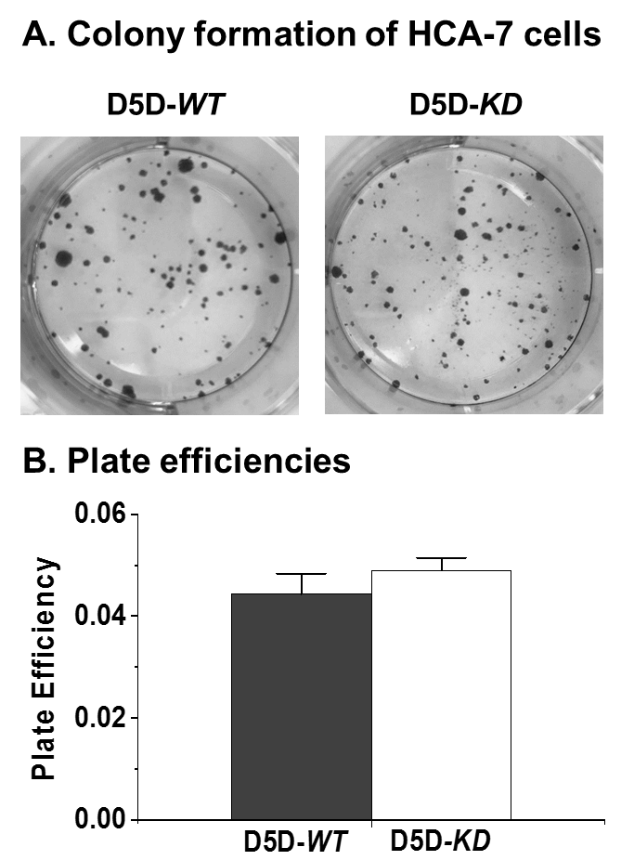
**

**Supplemental Figure 1.** D5D-*KD* alone did not affect HCA-7 cell growth. **A.** Colony formation of D5D-*WT* and D5D-*KD* HCA-7 cells at 10 days without DGLA treatment. **B.** Calculated plate efficiencies (*i.e.* total number of colonies counted/total number of cells seeded).
