# Supplementary material for: Dihomo-γ-linolenic acid inhibits xenograft tumor growth in mice bearing shRNA-transfected HCA-7 cells targeting delta-5-desaturase
Source: BMC Cancer. 2018 Dec 19;18:1268. doi: 10.1186/s12885-018-5185-9 (PMC6299961; doi:10.1186/s12885-018-5185-9)
Supplement: Supplementary file 3 — Table S2. Statistical analysis (ANOVA) for tumor size in Fig. 4b. The four groups are: Group 5: D5D-KD tumor control; Group 6: D5D-KD tumor w/DGLA; Group 7: D5D-KD tumor w/5-FU; Group 8: D5D-KD tumor w/DGLA and 5-FU. The six inserts (left to right from first row to second row) in each cell represents the statistics data at 10, 14, 17, 21, 24 and 28 days after treatment, respectively. *: significance with p < 0.05, **: significance with p < 0.01. (DOCX 16 kb) [file 12885_2018_5185_MOESM3_ESM.docx]

**Supplemental Table 2**

|  | Group 5 | Group 6 | | | Group 7 | | | Group 8 | | |
| --- | --- | --- | --- | --- | --- | --- | --- | --- | --- | --- |
| Group 5 | N/A | * | * | ** | ** | ** | ** | ** | ** | ** |
|  |  | ** | ** | ** | ** | ** | ** | ** | ** | ** |
| Group 6 |  | N/A | | | - | - | - | - | - | * |
|  |  |  |  |  | - | - | - | * | * | ** |
| Group 7 |  |  | | | N/A | | | - | - | - |
|  |  |  |  |  |  |  |  | - | - | - |
| Group 8 |  |  | | |  | | | N/A | | |

**Supplemental Table 2.** Statistical analysis (ANOVA) for tumor size in Fig 4B. The four groups are: **Group 5:** D5D-*KD* tumor control; **Group 6:** D5D-*KD* tumor w/DGLA; **Group 7:** D5D-*KD* tumor w/5-FU; **Group 8:** D5D-*KD* tumor w/DGLA and 5-FU. The six inserts (left to right from first row to second row) in each cell represents the statistics data at 10, 14, 17, 21, 24 and 28 days after treatment, respectively. *: significance with p < 0.05, **: significance with p < 0.01.
