# Supplementary material for: Dihomo-γ-linolenic acid inhibits xenograft tumor growth in mice bearing shRNA-transfected HCA-7 cells targeting delta-5-desaturase
Source: BMC Cancer. 2018 Dec 19;18:1268. doi: 10.1186/s12885-018-5185-9 (PMC6299961; doi:10.1186/s12885-018-5185-9)
Supplement: Supplementary file 4 — Table S3. Two factor experiment table of the additive effect from D5D-KD/DGLA along with 5-FU’s on HCA-7 xenograft tumor growth. Measured tumor size after 4-week treatment from (a) mice with D5D-WT tumor after vehicle treatment, (b) mice with D5D-WT tumor after 5-FU treatment, (c) mice with D5D-KD tumor after DGLA supplementation, and (d) mice with D5D-KD tumor after combination of DGLA and 5-FU treatment. Data represent mean ± SD with six mice per groups. (DOCX 15 kb) [file 12885_2018_5185_MOESM4_ESM.docx]

**Supplemental** **Table 3**

|  | **w/o 5-FU** | **w/ 5-FU** |
| --- | --- | --- |
| **w/o D5D-*KD*/DGLA** | ^a^306.6 ± 65.4 mm^3^ | ^b^204.3 ± 55.3 mm^3^ |
| **w/ D5D-KD/DGLA** | ^c^178.2 ± 31.9 mm^3^ | ^d^100.1 ± 24.3 mm^3^ |

**Supplemental** **Table 3.** Two factor experiment table of the additive effect from D5D-*KD*/DGLA along with 5-FU’s on HCA-7 xenograft tumor growth. Measured tumor size after 4-week treatment from (a) mice with D5D-*WT* tumor after vehicle treatment, (b) mice with D5D-*WT* tumor after 5-FU treatment, (c) mice with D5D-*KD* tumor after DGLA supplementation, and (d) mice with D5D-*KD* tumor after combination of DGLA and 5-FU treatment. Data represent mean ± SD with six mice per groups.
