# Supplementary material for: Dihomo-γ-linolenic acid inhibits xenograft tumor growth in mice bearing shRNA-transfected HCA-7 cells targeting delta-5-desaturase
Source: BMC Cancer. 2018 Dec 19;18:1268. doi: 10.1186/s12885-018-5185-9 (PMC6299961; doi:10.1186/s12885-018-5185-9)
Supplement: Supplementary file 5 — Figure S2. Body weight of mice bearing HCA-7 xenograft tumors during 4-week treatment. A. Measured body weight of mice bearing D5D-WT tumors during 4-week treatment. B. Measured body weight of mice bearing D5D-KD tumors during 4-week treatment. (DOCX 71 kb) [file 12885_2018_5185_MOESM5_ESM.docx]

**Supplemental Figure 2**


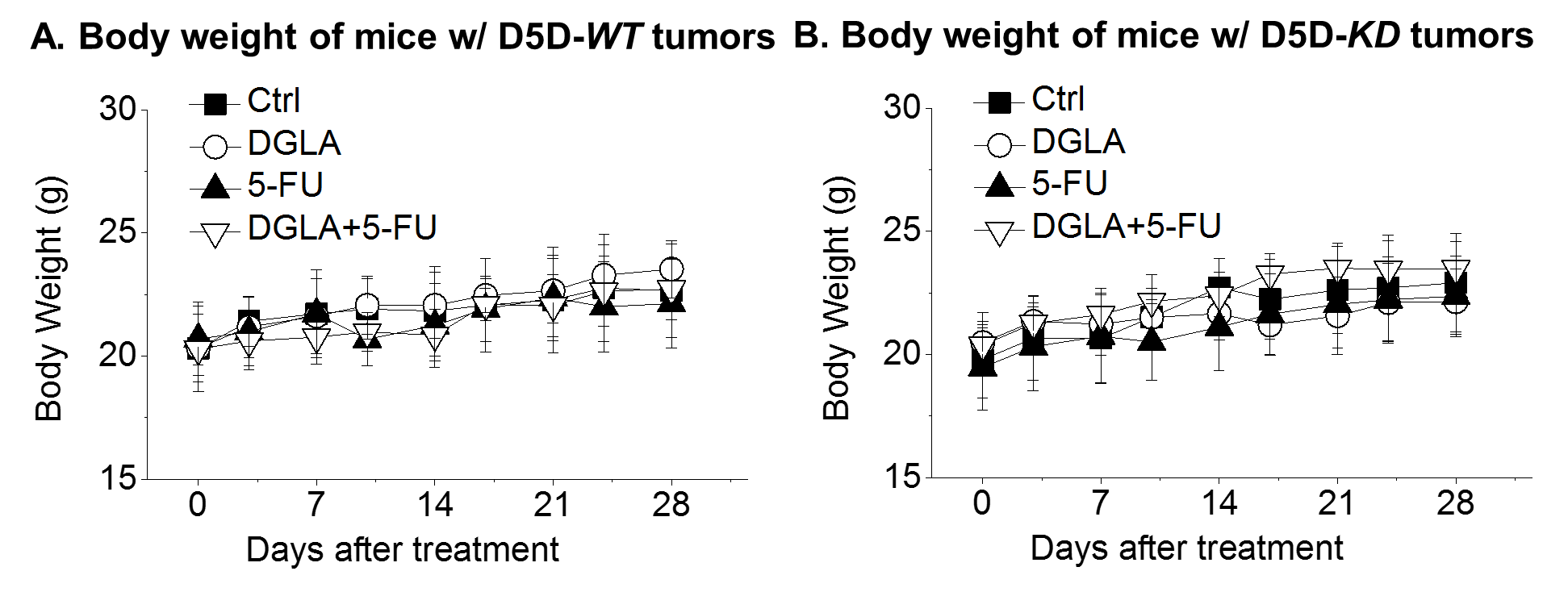


**Supplemental Figure 2.** Body weight of mice bearing HCA-7 xenograft tumors during 4-week treatment. **A.** Measured body weight of mice bearing D5D-*WT* tumors during 4-week treatment**. B.** Measured body weight of mice bearing D5D-*KD* tumors during 4-week treatment**.**
